# Hawthorn Proanthocyanidin Extract Inhibits Colorectal Carcinoma Metastasis by Targeting the Epithelial-Mesenchymal Transition Process and Wnt/β-Catenin Signaling Pathway

**DOI:** 10.3390/foods13081171

**Published:** 2024-04-12

**Authors:** Ziwei Wang, Yasai Sun, Mengying Wu, Liangfu Zhou, Yu Zheng, Ting Ren, Meijiao Li, Wen Zhao

**Affiliations:** College of Food Science and Technology, Hebei Agricultural University, Baoding 071001, China; wzw17861313227@163.com (Z.W.); sunyasai@hebau.edu.cn (Y.S.); wumy9902@163.com (M.W.); zhoulf202201@163.com (L.Z.); zhengyu9818@163.com (Y.Z.); rt1134771972@163.com (T.R.); 20212070138@pgs.hebau.edu.cn (M.L.)

**Keywords:** hawthorn proanthocyanidin, colorectal carcinoma cells, network pharmacology, Wnt/β-catenin signaling pathway

## Abstract

Colorectal carcinoma (CRC) is a major global health concern, with cancer metastasis being the main cause of patient mortality, and current CRC treatments are challenged by drug resistance. Although natural compounds, especially in foods like hawthorn proanthocyanidin extract (HPOE), have good anticancer activity, their effects on CRC metastasis remain unknown. Therefore, our objective was to investigate the impact and potential mechanisms of HPOE on the movement and infiltration of cells in the HCT116 CRC cells. Firstly, scratch-healing experiments confirmed the anti-migratory and anti-invasive capabilities of HPOE. Then, network pharmacology identified 16 possible targets, including MMP-9. Subsequently, RT-qPCR and Western blotting experiments confirmed that HPOE downregulated epithelial-mesenchymal transition-related factors (N-cadherin and MMP-9) and inhibited Wnt/β-catenin pathway activation. Finally, these results were experimentally validated using the Wnt pathway activator Licl and inhibitor XAV939. It was confirmed that HPOE had a certain inhibitory effect on the activation of the Wnt signaling pathway caused by the activator Licl and could enhance the inhibitory effect of the inhibitor XAV939. Our findings provide a basis for developing functional foods or dietary supplements, especially positioning HPOE as a functional food raw material for adjuvant treatment of CRC, given its ability to inhibit metastasis through the Wnt/β-catenin pathway.

## 1. Introduction

Colorectal carcinoma (CRC) stands as the second largest prevalent malignancy in the world [1]. Over the past few years, there has been a decrease in the overall prevalence of CRC, but the occurrence and fatality rates among patients under 50 years old are rising [2,3]. In patients with CRC, metastasis remains the primary cause of death, whereas CRC recurrence is predominantly attributed to residual tumor cells that had metastasized to distant organs before surgery [4]. Addressing the synchronization and metachronous recurrence in distant organs is the main challenge in the treatment of CRC [5]. Recent years have witnessed considerable advancements in traditional chemotherapy, radiotherapy, and targeted therapy for CRC. However, the prognosis for advanced CRC is unfavorable, and current treatment methods are ineffective in preventing cancer recurrence and metastasis, attributed to conditions such as drug resistance and low response rate [6,7]. Natural active ingredients in plants, such as anthocyanins, have the advantages of low cytotoxicity and strong antioxidant activity, and play a useful role in the prevention and relief of CRC [8]. Therefore, it is necessary to find natural active ingredients to assist chemotherapeutic drugs to effectively play the role of anti-recurrence and metastasis of CRC.

Some studies have highlighted the benefits of increased fruit and vegetable consumption in CRC. For example, *Pleurotus eryngii* powder consumption has been shown to enhance the survival rates of C26 CRC mice [9], and avocado extract from Mexico could induce the apoptosis of Caco-2 cells and reduce cancer risk [10]. These results suggest the potential of natural food-derived active ingredients as promising dietary supplements for the adjuvant treatment of CRC. Natural active ingredients in dietary plants can effectively inhibit the proliferation and metastasis of CRC by affecting CRC-related signaling pathways, such as Wnt/β-catenin, STAT1, STAT3, and MAPK [11]. For example, manuka honey inhibits the metastatic ability of CRC cells by regulating the expression of EMT-related markers such as β-catenin [12], and it is used as an adjuvant in the treatment of oral mucositis caused by radiation in cancer patients [13,14]. In addition, identifying drug targets in natural ingredients has gained prominence in innovative medical therapies for CRC. Individualized, targeted therapies have shown effectiveness in controlling CRC, a heterogeneous disease with various molecular characteristics, by directly inhibiting cell proliferation, differentiation, and migration, thereby improving patient prognosis [15,16]. Furthermore, network-based analysis offers various pharmacological targets [17,18]. Specifically, network pharmacology can elucidate potential interactions among multiple targets and active ingredients and identify potential targets for drug and disease therapies from a systems biology perspective [19], offering potential directions for natural active ingredients to target CRC.

Hawthorn, a medicinal and edible fruit, contains many natural bioactive substances, including polyphenols, flavonoids, and pentacyclic triterpenoids, demonstrating diverse biological activities [20]. For example, hawthorn extract has strong antioxidant activity [21] and has a good antibacterial effect on streptococcus mutans and candida albicans [22]. Hawthorn triterpenoid acid has a cholesterol-lowering effect by inhibiting intestinal cholesterol acyltransferase (ACAT) activity [23]. The versatility of hawthorn polysaccharides—recognized for their multiple benefits, including reducing blood lipid levels, anticancer, antibacterial, antioxidant, and immunomodulatory properties—in various health aspects has been previously demonstrated [24]. Hawthorn extract is beneficial to the inhibition of cancer. Studies have shown that phenylpropane derivatives isolated from hawthorn fruits have an effective anti-hepatoma effect [25]. Hawthorn polysaccharides could play an anticancer role by blocking the CRC cell cycle and inducing apoptosis [26]. Similarly, hawthorn kernel acetylated xylo-oligosaccharide has a certain inhibitory effect on the growth, migration, and invasion of CRC cells [27]. These findings indicate the significant potential of specific active components in hawthorn as promising dietary supplements against cancer. In addition, our earlier investigation revealed that hawthorn proanthocyanidin extract (HPOE) could inhibit the CRC cells’ cell cycle, inducing apoptosis and promoting macrovesicular death [28]. However, its effect on the metastasis of CRC cells remains unknown. Therefore, in this study, we first studied whether HPOE affected the migration and invasion of HCT116 cells by scratch-healing and transwell experiments, then we screened the key targets of HPOE inhibiting CRC by network pharmacology, predicted the possible anti-metastasis mechanism by gene ontology (GO) and Kyoto Encyclopedia of Gene Genomes (KEGG) enrichment, then preliminarily verified it by quantitative real-time PCR (RT-qPCR) and Western blotting, and further confirmed it by adding activators and inhibitors. The results of this investigation offer new perspectives for the research and development of functional food for the adjuvant treatment of CRC using HPOE.

## 2. Material and Methods

### 2.1. Materials

The materials used in the study included HCT116 cells (Suzhou Cas9x Biotechnology Co., Ltd., Suzhou, China) (The cell line comes from one of three malignant cell lines isolated and established in male CRC patients in 1979), Dulbecco’s modified Eagle medium (DMEM) (Beijing Solarbio Technology Co., Ltd., Beijing, China), phosphate-buffered saline (Seven Innovation Biotechnology Co., Ltd., Beijing, China), fetal bovine serum (FBS) (Beijing Quan jin Biotechnology Co., Ltd., Beijing, China), trypsin (Beijing Solarbio Technology Co., Ltd.), cell counting kit-8 (CCK-8) (Shanghai Bioscience Biotechnology Co., Ltd., Shanghai, China), Lithium Chloride (Licl) (Sigma-Aldrich Trading Co., Ltd. Shanghai, China), XAV 939 (MedChemExpress, Shanghai, China), 4% paraformaldehyde (Beyotime Biotechnology Co., Ltd., Shanghai, China), Giemsa stain solution (Beijing Solarbio Technology Co., Ltd.), Matrigel (Beijing Solarbio Technology Co., Ltd.), phosphorylase inhibitor (Seven Innovation Biotechnology Co., Ltd.), RIPA lysis buffer (Jiangsu Cowin Biotech Co., Ltd., Taizhou, China), seven-color protein marker Ⅱ (10–180 kDa) (Seven Innovation Biotechnology Co., Ltd.), Omni-Easy^TM^ one-step page gel fast preparation kit (Shanghai Ya Enzyme Biotechnology Co., Ltd., Shanghai, China), Western blocking buffer (bovine serum albumin, BSA) (Seven Innovation Biotechnology Co., Ltd.), and NcmECL Ultra (New Cell and Molecular Biotech Co., Ltd., Suzhou, China).

### 2.2. HPOE Extraction

Hawthorn fruit was extracted with ethanol (Solarbio, China) at 80 °C to obtain the crude extract of hawthorn proanthocyanidins, and then HPOE was obtained by separation and purification with ethyl acetate and AB-8 macroporous resin (Solarbio, China) [29].

### 2.3. Cell Lines and Culture

HCT116 cells were cultured in 10% FBS and 100 U/mL penicillin-streptomycin DMEM at 5% CO_2_ and 37 °C. Each T-25 flask contained 7 mL of culture medium.

### 2.4. Cell Viability Assay

Cells in the logarithmic growth stage were seeded at a density of 7 × 10^3^ cells/well in 96-well plates and cultured for 24 h. Control and drug-administered groups were established. After cell adhesion, different concentrations of HPOE (0, 100, 150, 200, 250, 300, 350, and 400 μg/mL), the Wnt signaling pathway activator Licl (0, 2.5, 5, 10, 20, 40, 80, and 160 μM), and the Wnt signaling pathway inhibitor XAV939 (0, 2.5, 5, 7.5, 10, 12.5, 15, 17.5, and 20 μM) were administered for 24 and 48 h. Then, the CCK-8 solution was mixed with serum-free medium at a ratio of 1:10 *v*/*v*, adding 100 μL per well. The cells were then cultured in a 5% CO_2_ incubator at 37 °C for 1–3 h. Finally, the absorbance of each well at 450 nm was measured using spectrophotometer. Each experiment was conducted in triplicate.

### 2.5. Cell Treatment Groups

In the cell viability assay, we found that the IC50 value of HCT116 cells cultured with HPOE for 48 h was 299.76 μg/mL, and the cell viability of the 10 μM Licl group increased significantly. Combined with SunKang et al., 10 μM was used as the XAV939 dose to culture CRCSW480 cells for experiments [30], and HCT116 cells in the logarithmic phase were divided into the following groups: control, HPOE 200 μg/mL, HPOE 250 μg/mL, HPOE 300 μg/mL, Licl (10 μM), HPOE + Licl (HPOE 250 μg/mL + Licl 10 μM), XAV939 (10 μM), and HPOE + XAV939 groups (HPOE 250 μg/mL + XAV939 10 μM).

### 2.6. Scratch-Healing Assay

HCT116 cells (4 × 10^5^ cells/well) were inoculated into six-well plates and subjected to scratch healing when they reached 100% confluence. A sterile 200 μL pipette tip was used to create cell-free scratches on the cell monolayer [31]. After discarding the medium and washing off impurities with phosphate-buffered saline, 2 mL of the corresponding treatment solutions were added to each well, and the culture was continued for 48 h. Scratch healing was observed and photographed at 0 and 48 h after scratching using a 40× microscope. The scratch-healing rates were calculated as follows: rate (%) = (0–48 h scratch width)/0 h scratch width × 100%.

### 2.7. Transwell Migration and Invasion Assay

Transwell assay was conducted to assess the migration and invasion capacities of HCT116 cells [32]. Following drug pretreatment, the cells (1 × 10^5^ cells/well) were resuspended in 200 μL of DMEM without serum and then positioned in the upper chamber. The lower chamber was filled with 600 μL of DMEM that comprised 10% FBS. Following an incubation period of 24 h, the non-migrating cells in the upper chamber were gently wiped off, and then the transwell chamber was fixed with 4% paraformaldehyde for 30 min and stained with 1% Giemsa stain solution for 30 min. For the transwell invasion assay, 66 μL of Matrigel, diluted 1:2.3 with serum-free medium, was added to each well before cell inoculation on the membrane at 37 °C for 30 min. Subsequent steps mirrored those of the cell migration experiments. Each group was subjected to three independent experiments, and the number of migrated or invaded cells was statistically analyzed.

### 2.8. Target Screening of HPOE

Through LC-ESI-MS analysis, our earlier findings showed that HPOE primarily consists of epicatechin and proanthocyanidins B2, B5, and C1 [33]. The structural information and related targets of these active components were determined through the TCMSP (https://tcmsp-e.com/) (accessed on 19 May 2023) and Pub Chem (https://pubchem.ncbi.nlm.nih.gov/) (accessed on 19 May 2023) databases. Then, the identified structures were imported into the Swiss Target Prediction online platform (http://www.swisstargetprediction.ch/) (accessed on 19 May 2023), UniProt database (https://www.uniprot.org/id-mapping) (accessed on 20 May 2023), and the DrugBank database (https://dev.drugbank.com/guides/drugbank/searching) (accessed on 20 May 2023) to give the target prediction and integrate the results.

### 2.9. Construction of Component–Target Networks

The network between the active components of HPOE and their potential targets was constructed by Cytoscape 3.9.1 software. The value of the participating interaction protein (degree) was obtained by the Network Analyzer tool. The evaluation of network node centrality involved assessing various topology parameters, including degree centrality, betweenness centrality, and closeness centrality. A node’s degree centrality increased with its higher degree, signifying greater importance within the network [34].

### 2.10. Screening of Disease Targets for CRC

Using the keywords “colon cancer” and “CRC”, the targets associated with CRC were searched for in the DisGeNET (http://www.disgenet.org/home/) (accessed on 23 May 2023), OMIM (https://omim.org/) (accessed on 23 May 2023), and GeneCards (https://www.genecards.org/) (accessed on 23 May 2023) databases. “Score_gda” > 0.1 was used as the screening condition for the DisGeNET database, and a relevance score of ≥10 was used as the screening standard for the GeneCards database. These candidate targets were reintegrated as potential targets for preventing and treating CRC using HPOE.

### 2.11. Construction of Protein–Protein Interaction Networks

The active ingredient and CRC-related targets were intersected, generating a list of common targets, which were visualized through Venn diagrams. The intersection targets were then uploaded into the STRING platform (https://string-db.org/) (accessed on 25 May 2023), specifying “*Homo sapiens*” as the organism type and setting a minimum interaction threshold of 0.4 to obtain a protein–protein interaction (PPI) file. Then, the generated PPI file was imported into Cytoscape 3.9.1 to generate a map of the PPI network and the Network Analyzer tool was used for topological analysis. Key genes were identified through screening with the CentiScaPe 2.2 Menu [35].

### 2.12. GO Enrichment and KEGG Pathway Analyses

The DAVID bioinformatics database (https://david.ncifcrf.gov/) (accessed on 20 June 2023) functional annotation tool was used for the enrichment analysis of core targets, and the results of GO enrichment analysis were obtained, encompassing biological processes (BPs), cellular components (CCs), and molecular functions (MFs). Using the parameters of count ≥ 2 and *p*-value < 0.05, the top 15 GO items were selected, and a bar chart was drawn using the Micro Sheng Letter platform (http://www.bioinformatics.com.cn) (accessed on 29 June 2023) for visual analysis. Similarly, KEGG pathway enrichment analysis of the core targets was carried out using the DAVID database, and under the condition of count ≥ 2 and *p* value < 0.05, we established the top 20 KEGG pathways that demonstrated significant enrichment and a bubble map was drawn for visual analysis.

### 2.13. RNA Extraction and RT-qPCR

Total RNA from HCT116 cells was isolated using a TRIzol reagent (Ambion, Shanghai, China). After 48 h of culture with the corresponding drug treatments, the cells were collected, and the total RNA was extracted with 1 mL of TRIzol reagent. Reverse transcription was performed after adjusting the RNA concentration based on the guidelines provided by the PrimeScript^TM^RT Reagent Kit with gDNA Eraser (TakaraBio, Beijing, China) [36]. According to the instructions of TB Green^®^ Premix Ex Taq™ Ⅱ kit, using the primer sequence synthesized by General Biology (Anhui) Co., Ltd. (Chuzhou, China), the PCR reaction was carried out with GADPH as an internal reference. Table 1 shows the primer sequences used in this study. The target gene mRNA expression level was calculated using the 2^−ΔΔCt^ method.

### 2.14. Western Blotting

The HCT116 cells were exposed to different amounts of HPOE (0, 200, 250, and 300 μg/mL), 250 μg/mL HPOE + 10 μM Licl, or 250 μg/mL HPOE + 10 μM XAV939 for 48 h. After trypsin digestion, cells from the different groups were collected, and total protein was extracted by a RIPA lysis buffer. A Bradford protein assay kit (Beijing Leagene Biotechnology Co., Ltd., Beijing, China) was used to determine the protein concentration. Protein samples were isolated using 10% SDS-PAGE and transferred onto a polyvinylidene fluoride (PVDF) membrane (PALL Company, Washington, NY, USA), which was then sealed with BSA. Subsequently, the membranes were incubated with primary antibodies overnight at 4 °C, followed by incubation with the corresponding secondary antibodies. Target protein bands were visualized using a chemiluminescence kit. Gray values were analyzed using ImageJ 1.54f software [37]. The primary antibodies (Wuhan Sanying Biology Technology Co., Ltd., Wuhan, China) used in this study included E-cadherin (1:8000), N-cadherin (1:5000), matrix metalloproteinase (MMP)-9 (1:800), β-actin (1:5000), β-catenin (1:8000), c-Myc (1:8000), Snail (EPR21043, Abcam Trading Co., Ltd., Shanghai, China) (1:1000), and corresponding secondary antibodies (1:5000). Each group has three samples.

### 2.15. Statistical Analysis

The data are presented as mean ± standard deviation. After a normality test, Student’s *t* test was used to analyze the differences between groups. The significance level was set at *p* < 0.05. Statistical analysis was conducted using Origin 2018 software.

## 3. Results

### 3.1. HPOE Inhibits HCT116 Cells Migration and Invasion

**Figure 1:**
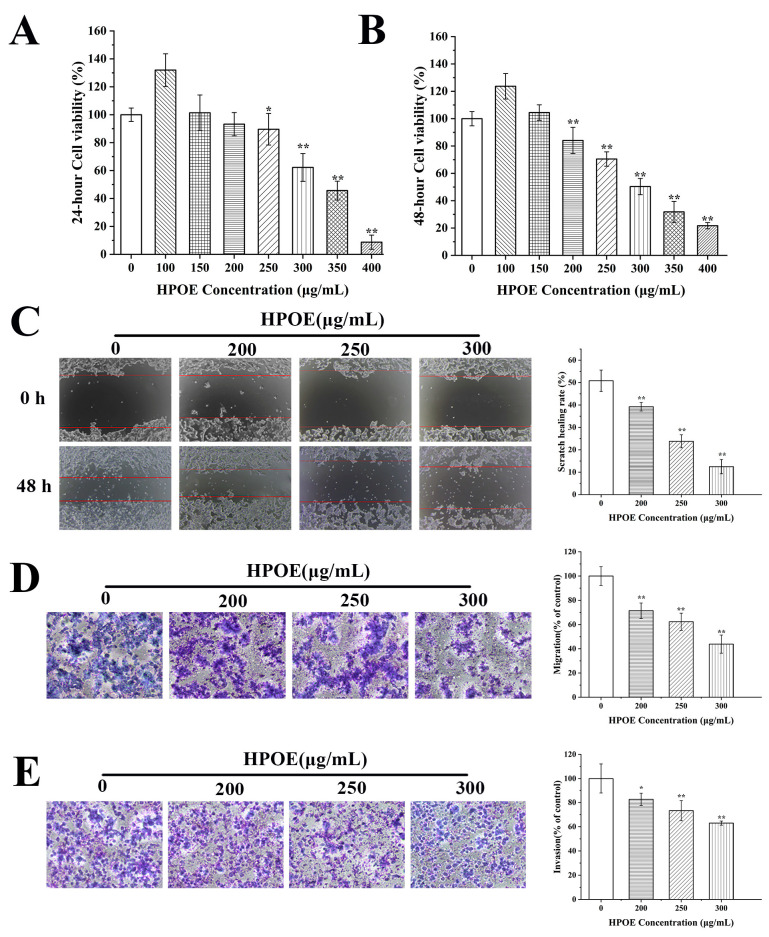
Effect of HPOE on migration and invasion of HCT116 cells. (**A**) Cell viability of HCT116 cells treated with HPOE for 24 h; (**B**) cell activity of HCT116 cells treated with HPOE for 48 h; (**C**) the effect of HPOE on the migration ability of HCT116 cells was detected through a scratch test (×40), the edges of scratches were marked with red lines; (**D**) the transwell migration assay was used to detect the effect of HPOE on the migration ability of human HCT116 cells; (**E**) the transwell invasion assay was used to detect the effect of HPOE on the invasive ability of HCT116 cells. HPOE, hawthorn procyanidins extract. * *p* < 0.05; ** *p* < 0.01, compared with the blank control group.

The HCT116 cells were subjected to various concentrations of HPOE (0–400 μg/mL) in order to assess the impact on their inhibitory properties (Figure 1A,B). The results indicated a noteworthy decline in the viability of the HCT116 cells when exposed to HPOE, and it was observed that the IC50 values at 24 and 48 h were 321.64 μg/mL and 299.76 μg/mL, respectively. Treatment with 200 μg/mL HPOE for 24 h exhibited a modest effect on cell viability, intensifying significantly after 48 h. Moreover, the cell survival rate decreased significantly to 31.84% after exposure to treatment with 350 μg/mL HPOE for 48 h, accompanied by a substantial increase in the cell death rate. Therefore, we selected a treatment duration of 48 h and HPOE concentrations of 200, 250, and 300 μg/mL for follow-up experiments. 

We then investigated the effects of HPOE on HCT116 cells’ migration and invasion through scratch-healing and transwell experiments. Our findings revealed that HPOE significantly inhibited HCT116 cells’ migration. The scratch-healing rate of HCT116 cells treated with 300 μg/mL HPOE was 38.35% lower than that in the control group (50.82%) (Figure 1C). At the same time, the migration rates ranged from 43.85% to 71.46% after treatment with 200–300 μg/mL HPOE, which was significantly lower than that of the control group (Figure 1D). In addition, HPOE has a certain inhibitory effect on the invasive ability of HCT116 cells. When the concentration of HPOE was 200–300 μg/mL, the number of invasive cells decreased significantly. The invasion rate ranged from 62.97 to 82.60% (Figure 1E). These findings confirm that HPOE inhibits CRC cell migration and invasion.

### 3.2. Prediction of Potential Targets and Mechanisms Underlying the HPOE-Mediated Inhibition of CRC Metastasis Based on Network Pharmacology

#### 3.2.1. Target Analysis of the Active Components of HPOE

**Figure 2:**
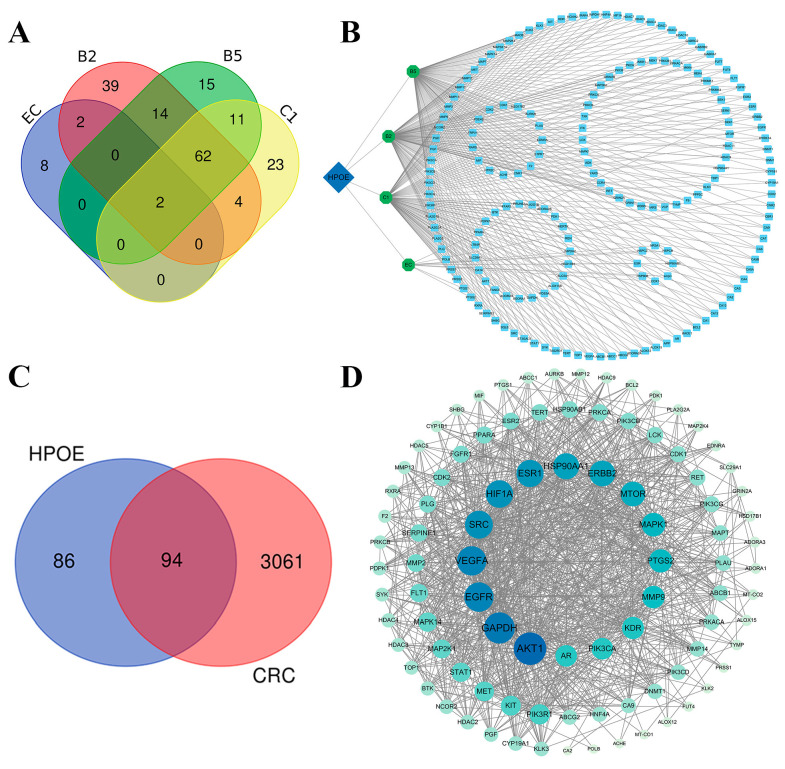
HPOE and CRC target prediction and network analysis. Venn diagram (**A**) and component–target diagram of HPOE active components (**B**). Venn diagram of HPOE and CRC-related targets (**C**) and protein–protein interaction (PPI) network (**D**). The diamond represents HPOE, the oval denotes several compounds of HPOE, and the square represents the predicted target. In the PPI network, the nodes with darker colors and larger diameters indicate a stronger correlation with other targets, representing the interaction between the targets. HPOE, hawthorn procyanidins extract; CRC: colorectal carcinoma.

Based on the four main active components of EC and proanthocyanidins B5, B2, and C1, we collected 341 potential targets of HPOE from target databases (TCMSP, Pub Chem, Swiss Target Prediction, and DrugBank database). These comprised 12 targets of EC, 104 for proanthocyanidin B5, 123 for proanthocyanidin B2, and 102 for proanthocyanidin C1. After removing duplicate targets, 180 unique targets were obtained for subsequent analyses. The Venn diagrams (http://bioinformatics.psb.ugent.be/webtools/Venn/) (accessed on 20 May 2023) showed that 95 targets exist between two or more HPOE components, and 85 targets exist in only one HPOE component (Figure 2A). To systematically elucidate the interaction between HPOE components and their corresponding targets, 341 potential targets of hawthorn procyanidins were utilized to construct a component–target network map using Cytoscape 3.9.1 software. The network comprised 185 nodes (5 composite nodes and 180 composite target nodes) and 690 edges (Figure 2B), with an average node value of 3.73. Proanthocyanidins B2 (degree = 124), B5 (degree = 105), and C1 (degree = 103) occupied key positions in this network. The results also showed that the four active components of HPOE targeted estrogen receptor 1 (ESR1) and cyclooxygenase 1 (PTGS1).

#### 3.2.2. CRC Target Prediction and PPI Network Analysis

Using “Colon cancer” and “CRC” as keywords, a total of 3155 CRC-related targets were obtained from the OMIM, DisGeNET, and GeneCards databases. By intersecting 180 HPOE targets with 3155 CRC-related genes and constructing a Venn diagram (Figure 2C), we identified 94 potential targets of HPOE in treating CRC. Subsequently, the STRING database was used to visually analyze these targets, and the corresponding PPI network was obtained (Figure 2D). The network had 94 nodes and 982 edges. The *p*-value of the PPI network enrichment was <1.0 × 10^−16^, suggesting a high level of credibility and strong correlation among the potential targets of HPOE when treating CRC. To further screen the key genes in the network further, 16 key target genes were identified using Cytoscape 3.9.1 software: v-akt murine thymoma viral oncogene homolog 1 (AKT1), glyceraldehyde-3-phosphate dehydrogenase (GAPDH), vascular endothelial growth factor (VEGFA), epidermal growth factor receptor (EGFR), steroid receptor coactivator (SRC), hypoxia inducible factor-1 (HIF1A), ESR1, heat shock protein 90 alpha family class A member 1 (HSP90AA1), epidermal growth factor receptor 2 (ERBB2), mechanistic target of rapamycin (MTOR), mitogen-activated protein kinase 1 (MAPK1), prostaglandin-endoperoxide synthase 2 (PTGS2), MMP-9, kinase insert domain receptor (KDR), androgen receptor (AR), and plasminogen (PLG). These targets may be attributed to the fundamental therapeutic effects of HPOE in CRC. Among these, MMP-9, known for degrading the extracellular matrix (ECM), plays a crucial role in cancer cell migration [38].

#### 3.2.3. GO Enrichment and KEGG Pathway Analyses

**Figure 3:**
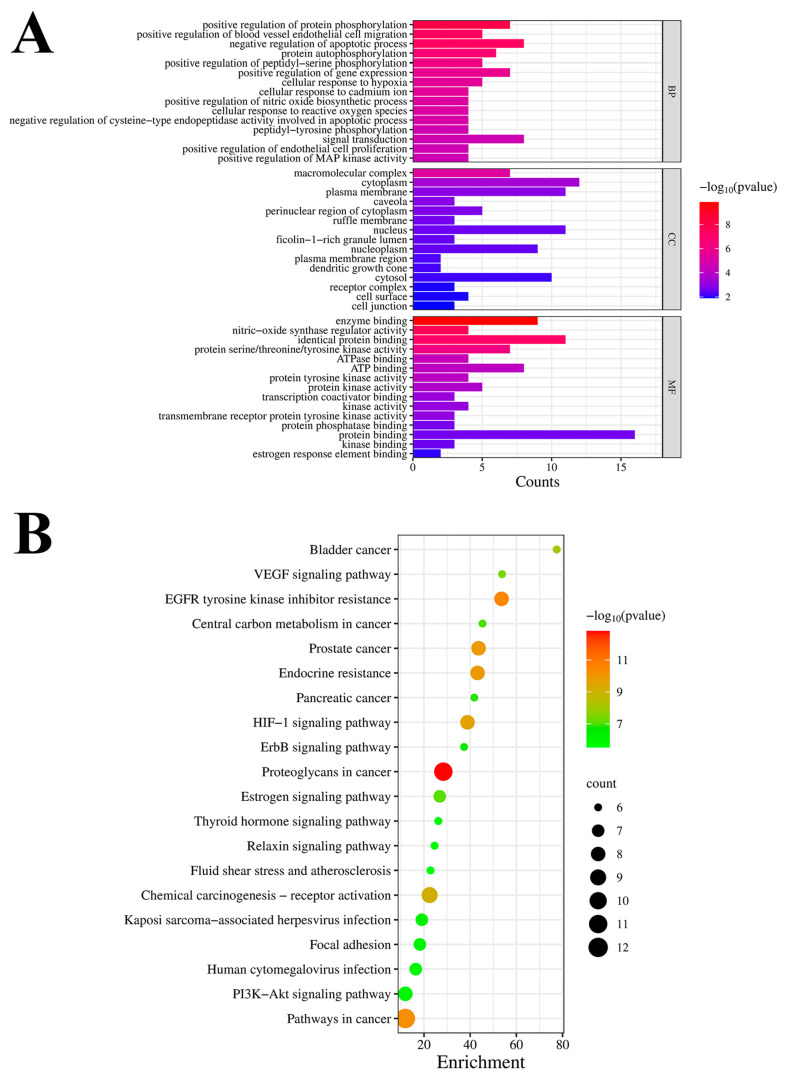
Enrichment analysis of GO terms and KEGG pathways in which HPOE may play a role in CRC. (**A**) GO analysis bar chart showing the top 15 terms, (**B**) KEGG bubble chart showing the top 20 pathways (*p* value < 0.05). BPs, biological processes; CC, cellular component; MF, molecular function.

In order to delve deeper into the possible routes of HPOE in CRC, the DAVID database was employed to analyze the 16 crucial target genes with regard to GO and KEGG pathway enrichment. The GO enrichment analysis yielded 225 GO terms (Figure 3A), including 154 terms related to BP, with a primary focus on the positive regulation of protein phosphorylation, positive regulation of blood vessel endothelial cell migration, negative regulation of apoptotic processes, protein autophosphorylation, and positive regulation of peptidyl-serine phosphorylation. Similarly, it included 29 MF terms, primarily focusing on enzyme binding, nitric oxide synthase regulator activity, identical protein binding, protein serine/threonine/tyrosine kinase activity, ATPase binding, ATP binding, and protein tyrosine kinase activity. Finally, there were 26 GO terms related to CC, predominantly enriched in the macromolecular complexes, cytoplasm, plasma membrane, and caveola. Furthermore, an analysis was performed to enrich the KEGG pathway, resulting in the identification of 100 signaling pathways. To illustrate the findings more effectively, a bubble chart was generated to showcase the top 20 signaling pathways (Figure 3B). These pathways were predominantly related to proteoglycans in cancer, EGFR tyrosine kinase inhibitor resistance, pathways in cancer, prostate cancer, endocrine resistance, and the HIF-1 signaling pathway, among others. Among them, proteoglycans and pathways in cancer constitute the majority of these pathways.

### 3.3. HPOE Inhibits the Expression of Genes and Proteins Related to EMT in HCT116 Cells

**Figure 4:**
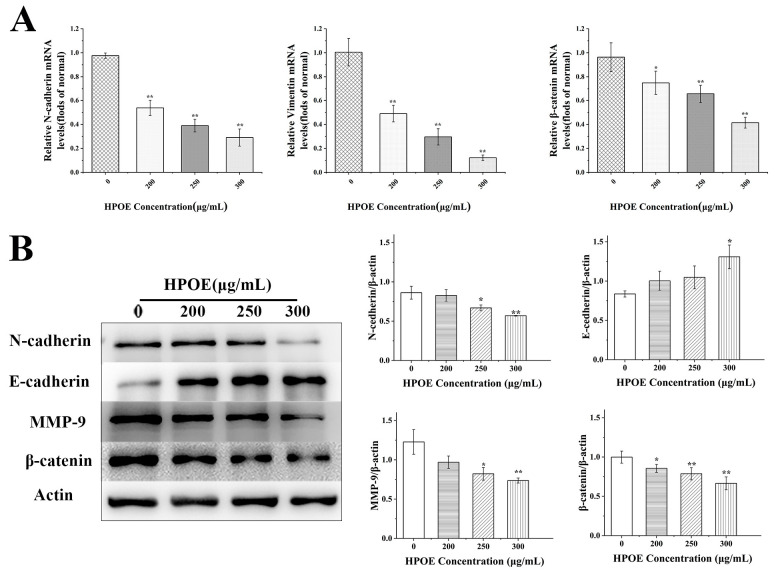
Expression of EMT-related molecules in HCT116 cells after HPOE treatment. (**A**) Expression of EMT-related mRNA; (**B**) expression of EMT-related protein. HPOE, hawthorn procyanidins extract; EMT, epithelial-mesenchymal transition. * *p* < 0.05, ** *p* < 0.01, compared with the blank control group.

After epithelial-mesenchymal transition (EMT) occurrence, the intercellular adhesion of cancer cells decreased, and the migration and invasion ability increased. Therefore, we investigated the effect of HPOE on genes’ expression associated with EMT in HCT116 cells by RT-qPCR. Compared to that in the untreated group, the mRNA expression levels of EMT-related molecules—*N-cadherin*, *Vimentin*, and *β-catenin*—significantly decreased in a concentration-dependent manner following HPOE treatment (Figure 4A). These findings were further verified using Western blotting. The results showed that after treating HCT116 cells with HPOE for 48 h, the high-dose HPOE group exhibited a significant upregulation in the expression of E-cadherin protein compared to the control group. Conversely, the expression levels of N-cadherin, MMP-9, and other proteins were significantly downregulated in the high-dose HPOE group (Figure 4B). Since β-catenin is both an EMT marker and a core factor of the Wnt signaling pathway [39], these findings strongly indicate that HPOE could inhibit EMT processes in HCT116 cells, which may be related to inhibiting the activation of the Wnt signaling pathway.

### 3.4. HPOE Inhibits the Expression of Genes and Proteins Related to the Wnt/β-Catenin Signaling Pathway in HCT116 Cells

**Figure 5:**
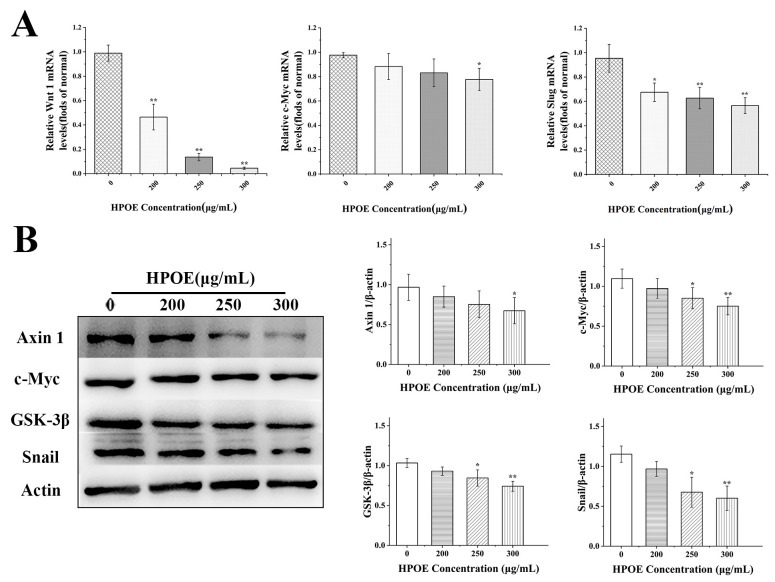
Expression of Wnt/β-catenin signaling pathway-related molecules in HCT116 cells after HPOE treatment. (**A**) Expression of mRNA related to Wnt/β-catenin signal pathway; (**B**) expression of proteins related to Wnt/β-catenin signaling pathway. HPOE, hawthorn procyanidins extract. * *p* < 0.05, ** *p* < 0.01, compared with the blank control group.

To determine whether HPOE inhibited CRC metastasis through the Wnt/β-catenin signaling pathway, we first used RT-qPCR to detect the effect of HPOE on the Wnt/β-catenin signaling pathway-related gene expression in HCT116 cells. In comparison to the blank group, the mRNA expression levels of *Wnt1*, *c-Myc*, and *Slug* decreased (Figure 5A). The Western blot analysis revealed a substantial downregulation in the levels of c-Myc, Snail, and other proteins upon a 48 h treatment of HCT116 cells to HPOE in the high-dose HPOE group (Figure 5B). These findings indicate that HPOE may inhibit cancer cell metastasis by inhibiting the activation of the Wnt/β-catenin signaling pathway.

### 3.5. HPOE Inhibits HCT116 Cells’ Migration and Invasion by Inhibiting the Wnt/β-Catenin Signaling Pathway

**Figure 6:**
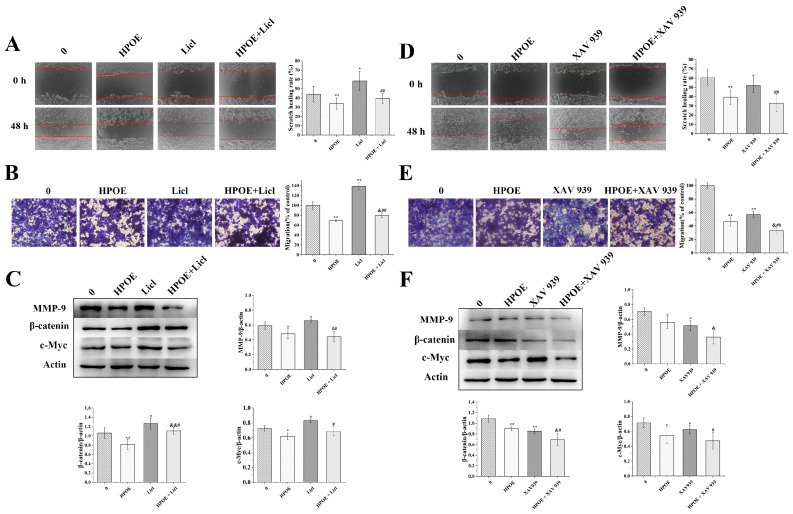
Effect of co-treatment of HPOE with Wnt signal pathway activator Licl or inhibitor XAV939 on HCT116 cells. (**A**) The effects of Wnt signal pathway activators Licl and HPOE on the migration of HCT116 cells were detected using a scratch test (×40), the edges of scratches were marked with red lines; (**B**) the transwell migration assay was used to detect the effect of Licl and HPOE on the migration ability of HCT116 cells; (**C**) expression of related proteins in HCT116 cells treated with Licl and HPOE; (**D**) effects of XAV939 and HPOE on the migration of HCT116 cells detected using a scratch test (×40), the edges of scratches were marked with red lines; (**E**) the transwell migration assay was used to detect the effect of XAV939 and HPOE on the migration ability of HCT116 cells; (**F**) expression of related proteins in HCT116 cells treated with XAV939 and HPOE. HPOE: hawthorn procyanidins extract. * *p* < 0.05, ** *p* < 0.01, compared with the blank control group; & *p* < 0.05, && *p* < 0.01, compared with HPOE group; # *p* < 0.05, ## *p* < 0.01, compared with Licl or XAV939 group.

These findings were further confirmed by additional experiments using Wnt signaling pathway activator and inhibitor. HCT116 cells were treated with different doses of the Wnt activator Licl (0–160 μM) and the Wnt inhibitor XAV939 (0–20 μM) for 48 h (Supplementary Figure S1). The cell survival rates for the activator groups treated with Licl 5 μM, 10 μM, and 20 μM were 104%, 109%, and 118%, respectively. Compared to that in the control group, the cell viability significantly increased in the 10 μM Licl group and was further enhanced in the 20 μM Licl group. Based on the experiment conducted by Sun Kang et al., a 10 μM XAV939 dose was used to treat CRC SW480 cells, so we chose a 10 μM XAV939 dose [30]. The activator Licl 10 μM was selected for the follow-up experiments. Further, the HCT116 cells were treated with 250 μg/mL HPOE, 10 μM Licl, and 10 μM XAV939 for 48 h. Subsequently, scratch-healing, transwell migration, and Western blot results showed that Licl significantly improved the migration ability of HCT116 cells and upregulated the expression of MMP-9, β-catenin, and c-Myc proteins. Compared with HPOE, Licl alleviated HPOE-induced migration inhibition to a certain extent, the number of HCT116 cells migrated significantly increased, and the expression of β-catenin protein was significantly upregulated (Figure 6A–C). In addition, XAV939 significantly inhibited HCT116 cells’ migration ability. It also significantly downregulated MMP-9, β-catenin, and c-Myc proteins’ expression, enhancing the inhibitory effect of HPOE on HCT116 cells’ migration (Figure 6D–F).

## 4. Discussion

CRC recurrence and metastasis significantly affect the prognosis of patients [40]. Current CRC treatment encounters challenges related to poor tolerance and severe side effects [7]. Therefore, prioritizing the development of methods that can effectively prevent, treat, and mitigate toxicity and side effects is imperative. Plant-derived natural active ingredients have demonstrated substantial potential in cancer treatment and prevention [41,42,43], especially those from natural foods. They have gained widespread use as functional and therapeutic foods, proving beneficial in CRC control or as auxiliary anticancer food support [44]. In the present study, HCT116 cells were treated with different concentrations of HPOE to observe their effects on cancer cell migration and invasion, and some possible mechanisms were verified using network pharmacology and experiments. We found that HPOE has a certain inhibitory effect on scratch-healing, migration and invasion, the Wnt/β-catenin signaling pathway, and the expression of EMT-related factors in HCT116 cells, thus inhibiting their metastasis.

CRC cells can be isolated from the primary lesion, spread through the blood and the circulatory systems, and transferred to distant organs such as the liver to form metastases [31,45]. This is related to the ability of CRC cells to migrate and invade. Therefore, we studied whether HPOE affects the migration and invasion of HCT116 cells. Compared to that in the control group, the scratch-healing ability of HCT116 cells was significantly inhibited, accompanied by a significant reduction in the number of cells migrating through the transwell insert to the lower chamber. This result suggests that HPOE may inhibit the matrix degradation of cancer cells, thereby reducing the invasive capability of CRC cells.

Utilizing network pharmacology, we employed a holistic approach based on systems biology to predict the potential mechanism of action of HPOE in inhibiting CRC [46,47]. Leveraging previous research on the components of HPOE, we identified potential targets for HPOE inhibition of CRC using disease and drug target databases. Subsequently, we constructed component–target data and protein interaction network diagrams, analyzing key targets’ biological processes and signaling pathways. This comprehensive approach allowed us to speculate on the potential mechanism underlying the efficacy of HPOE inhibiting CRC metastasis. Taking three parameters—degree, closeness, and betweenness—that surpassed the threshold values as the screening conditions, the PPI network of the 94 potential targets of HPOE in CRC treatment was analyzed, and 16 key targets such as AKT1, GAPDH, and MMP-9 were screened. These key targets were associated with biological processes such as positive protein phosphorylation regulation, positive vascular endothelial cell migration regulation, and negative apoptosis regulation. MMP-9 is an important member of the MMP family of proteins. MMPs are significant indicators of cancer prognosis that play a crucial role in ECM remodeling and the processing of bioactive molecules, they can degrade almost all matrix components and are closely associated with tumor angiogenesis, invasion, and metastasis [48,49]. Furthermore, KEGG pathway enrichment results highlighted that the potential targets of HPOE inhibition in CRC were primarily associated with both proteoglycans in cancer and pathways in cancer. Proteoglycans play key roles in regulating cell signal transduction and migration through interactions with extracellular ligands, growth factor receptors, ECM components, intracellular enzymes, and structural proteins [50]. In cancer, various proteoglycans influence tumor cell invasion, migration, and angiogenesis by regulating EMT, thereby affecting the initiation and progression of tumors [51,52,53]. In CRC, MMPs, particularly MMP-2 and MMP-9, are implicated in EMT, playing a role in the degradation of type IV collagen in the basement membrane and increasing tumor invasiveness [49]. This suggests that the inhibition of CRC metastasis by HPOE may be related to the influence of key targets like MMP-9 on EMT, thus inhibiting CRC cell migration and invasion.

EMT typically initiates metastasis during cancer progression, during which epithelial cells acquire migration and invasion characteristics, and MMPs’ expression increases, with the loss of E-cadherin and gain of N-cadherin and Vimentin [54,55,56]. β-catenin is an EMT marker and a core factor of the Wnt signaling pathway. Upon activation of Wnt, β-catenin undergoes translocation into the nucleus, inducing the expression of target genes such as MMPs, and inhibiting the expression of E-cadherin, which is beneficial to the development of EMT [31,39]. In our investigation, we observed that the expression of MMP-9, Vimentin, N-cadherin, and β-catenin decreased while that of E-cadherin increased. Various nutritious foods containing natural ingredients exhibit similar effects on CRC. For example, grape juice extract has shown the inhibition of MMP-2 and MMP-9 gene expression in CRC HT-29 and SW480 cells [57], and the natural selenium polysaccharide in *Pleurotus ostreatus* could upregulate the expression of E-cadherin and downregulate the expression of Vimentin in HCT116 cells and gastric cancer MGC-803 cells [58]. However, these studies only verified the expression of a few key regulatory proteins in the progression of EMT. Unlike this, our study not only included these contents but also explored the detailed mechanism of action and verified it by adding activators and inhibitors. In addition, since β-catenin is a core factor in the EMT and Wnt signaling pathways, its inhibition suggests that HPOE may inhibit the Wnt/β-catenin signaling pathway in HCT116 cells. Together, these findings suggest that HPOE could inhibit the EMT processes in HCT116 cells, affecting their migration and invasion capabilities, which may be related to inactivation of the Wnt signaling pathway.

Studies indicate that 93% of patients with CRC exhibit alterations in the Wnt signaling pathway, primarily involving the biallelic gene inactivation of adenomatous polyposis coli (APC) and mutations in catenin beta 1 (CTNNB1) [59]. Activation of the typical Wnt signal involves the Wnt ligand binding to Frizzed and low-density lipoprotein receptor-related protein 5/6. The β-catenin phosphorylation is impeded by this binding, resulting in the build-up of β-catenin within the cytoplasm. Subsequently, β-catenin migrates to the nucleus, binds to the T-cell Factor/Lymphoid Enhancing Factor (TCF/LEF) transcription factor, and activates the expression of genes associated with the Wnt signal [60,61]. In our study, the expression of the β-catenin protein, a key member of the Wnt/β-catenin signaling pathway, was downregulated. This downregulation may impede the binding of β-catenin with the TCF/LEF transcription factor, inhibiting the activation of downstream genes. This is supported by the downregulation of *Snail*, *c-Myc*, and other downstream target genes observed in this study. To further explore the correlation between the suppression of HPOE on the migration and invasion of CRC and the Wnt/β-catenin signaling pathway, we introduced Licl and XAV939 during cell culture. Licl can activate Wnt and stabilize free cytoplasmic β-catenin [62]. XAV939 can stabilize the β-catenin destruction complex, reduce β-catenin protein expression, and effectively inhibit the transmission of typical Wnt signals [63]. Our experimental results indicated that adding Licl weakened the migration-inhibitory effect of HPOE on HCT116 cells and concurrently upregulated the expression of MMP-9, β-catenin, and c-Myc proteins. Conversely, the addition of XAV939 augmented the migration-inhibitory effect of HPOE on HCT116 cells to some extent. Furthermore, it downregulated the expression of MMP-9, β-catenin, and c-Myc proteins.

In summary, this study initially verified the HPOE-mediated inhibition of CRC activity, migration, and invasion. Using network pharmacology, we made preliminary predictions about the anti-CRC targets and mechanism of HPOE, subsequently validating these predictions through further experimental research. The findings indicate that HPOE can inhibit the EMT of CRC cells, potentially inhibiting metastasis by promoting the inactivation of the Wnt/β-catenin signaling pathway. Next, we intend to further verify other ways of enrichment of KEGG.

## 5. Conclusions

In this study, scratch-healing and transwell experiments confirmed that HPOE had a certain inhibitory effect on the migration and invasion of HCT116 cells, and then we predicted by network pharmacology that the inhibition of HCT116 cell metastasis by HPOE may be related to MMP-9. RT-qPCR and Western blotting confirmed that HPOE could inhibit the EMT process related to MMP-9, and the further experimental results suggest that the inhibitory effect of HPOE in tumor metastasis may be related to the inhibition of EMT in cancer cells and the inactivation of the Wnt/β-catenin signal pathway. This implies that HPOE has the potential to be used as a dietary supplement in the treatment of CRC. in follow-up studies, we will explore the in vivo efficacy of HPOE against CRC. In addition, HPOE-related functional foods or dietary supplements also need to be further developed.

## Figures and Tables

**Table 1:** Primer sequences.

Gene	Forward (5′-3′)	Reverse (3′-5′)
*Wnt 1*	CGATGGTGGGGTATTGTGAAC	CCGGATTTTGGCGTATCAGAC
*Slug*	CGAACTGGACACACATACAGTG	CTGAGGATCTCTGGTTGTGGT
*N-cadherin*	TCAGGCGTCTGTAGAGGCTT	ATGCACATCCTTCGATAAGACTG
*c-Myc*	GGAGGCTATTCTGCCCATTTG	CGAGGTCATAGTTCCTGTTGGTG
*Vimentin*	AACCTGGCCGAGGACATCA	TCAAGGTCAAGACGTGCCAGA
*β-catenin*	CAACTAAACAGGAAGGGATGGA	CTATACCACCCACTTGGCAGAC
*GAPDH*	GTCAACGGATTTGGTCGTATTG	CTCCTGGAAGATGGTGATGGG

## Data Availability

The original contributions presented in the study are included in the article/Supplementary Materials, further inquiries can be directed to the corresponding author.

## Supplementary Materials

The following supporting information can be downloaded at https://www.mdpi.com/article/10.3390/foods13081171/s1. Supplementary Figure S1: Effects of Wnt signal pathway activator Licl and inhibitor XAV939 on the viability of HCT116 cells.
