# Supplementary material for: Hawthorn Proanthocyanidin Extract Inhibits Colorectal Carcinoma Metastasis by Targeting the Epithelial-Mesenchymal Transition Process and Wnt/β-Catenin Signaling Pathway
Source: Foods. 2024 Apr 12;13(8):1171. doi: 10.3390/foods13081171 (PMC11049232; doi:10.3390/foods13081171)
Supplement: Supplementary file 1 [file foods-13-01171-s001.zip › foods-2901862-supplementary.pdf]

Supplementary Figure

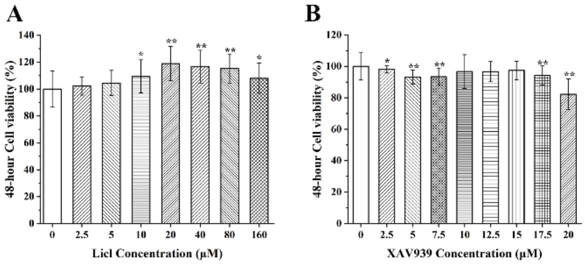

Supplementary Figure S1. Effects of Wnt signal pathway activator Licl and inhibitor XAV939 on the viability of HCT116 cells. (**A**) Cell viability of HCT116 cells treated with Licl for 48 h; (**B**) cell viability of HCT116 cells treated with XAV939 for 48 h. * $P < 0.05$; ** $P < 0.01$. Compared with the blank control group.
